# Prevalence, Risk Factors, and Patient Awareness of Diabetic Retinopathy in Saudi Arabia: A Review of the Literature

**DOI:** 10.7759/cureus.11991

**Published:** 2020-12-09

**Authors:** Abdullah Mohammed D Alharbi, Abdullah Masad S Alhazmi

**Affiliations:** 1 College of Medicine, Taibah University, Medina, SAU

**Keywords:** diabetes mellitus, diabetic retinopathy, diabetic macular edema, risk factors for diabetic retinopathy, saudi arabia, prevalence, awareness

## Abstract

Diabetes mellitus is a common disease in Saudi Arabia and globally, with some studies reporting about 30% prevalence among the Saudi population. Diabetic retinopathy (DR) is the leading cause of blindness among working-age patients, and it has a prevalence of 34.6% among diabetics worldwide. The objectives of this review were to examine the prevalence of DR in Saudi Arabia, describe the major associated risk factors, and raise awareness about the disease among diabetics.

The prevalence of DR in Saudi Arabia has risen substantially in recent decades. Significant risk factors associated with DR include older age, longer duration of diabetes, poor glycemic control, and hypertension. Awareness and education about diabetes are associated with better outcomes and fewer complications.

## Introduction and background

Diabetes mellitus is a metabolic disorder that results from disturbances in insulin secretion, insulin action, or both, and leads to chronic hyperglycemia with defects in carbohydrate, lipid, and protein metabolism, as defined by the World Health Organization (WHO) [1]. Estimates of the worldwide prevalence of diabetes mellitus in 2014 were 9% in men and 7.9% in women, which were nearly double the 1980 figures of 4.3% and 5% in men and women respectively [2], which is reflective of an increase in risk factors like obesity and poor lifestyle choices. This trend is related to the recent economic spurt in Saudi Arabia, which has brought with it major lifestyle changes with unhealthy eating habits and lack of exercise becoming the norm. As a result, the prevalence of diabetes mellitus has skyrocketed, with 23.9% of the total Saudi population affected by it [3]. Globally, 1.5 million deaths were attributed to diabetes mellitus in 2012, making it the eighth leading cause of death in that year [4].

Among the many complications of diabetes mellitus, diabetic retinopathy (DR) is a leading cause of blindness in patients aged 20-74 years [5], with a prevalence of 34.6% among diabetic patients globally [6]. Major risk factors for DR include longer duration of diabetes, higher levels of HbA1c, and high blood pressure [6]. The course of the disease starts with a mild nonproliferative stage characterized by the presence of microaneurysms only and progresses eventually to proliferative DR with neovascularization and vitreous or preretinal hemorrhage [7].

The pathophysiology of DR is not well understood, but a number of theories have been postulated. The most probable among them is the intraocular formation of reactive oxygen species (ROS) and the activation of protein kinase C that causes hyperpermeability of the retinal vasculature and the subsequent alterations in retinal blood flow. Protein kinase C also causes thickening of the basement membrane, leading to ischemia and cellular signaling by vascular endothelial growth factors (VEGFs) causing ocular neovascularization [8].

Even though the early treatment of DR could reduce severe vision loss by 90% [7], there are some factors that pose difficulty for patients to seek the care they need. These include lack of awareness and cost of treatment [9]. Barriers to screening for DR were found to be more common in diabetic patients with lower socioeconomic status; depression, financial problems, and transportation issues were the most commonly identified barriers [10].

## Review

Prevalence of diabetic retinopathy

In a study that was published in 1986 investigating the most common causes of blindness and visual impairment in Saudi Arabia [11], it was concluded that the prevalence of blindness (visual acuity of <3/60) among Saudis was 1.5% and that of visual impairment (visual acuity of <6/18 and ≥3/60) was 7.8%; the leading cause of blindness was cataracts, accounting for 52.8% of cases with an overall prevalence of 42.4 per 1,000 people. DR, on the other hand, along with pigmentary degeneration and other non-congenital retinal diseases, was classified as "retinal lesions" and only had a collective prevalence of 3.3 per 1,000 people. The reason behind these low figures could be attributed to the fact that around 50% of participants were younger than 15 years of age. However, age groups of 40-59 years and above 60 years had a prevalence of 8.9 per 1,000 and 27.1 per 1,000 respectively [11]. These figures, however, might be inflated since one of the limitations of this study was that it was carried out by examining participants in their homes during the daytime when men fit for work were usually working outside of their homes; but these figures still clearly show the very low prevalence of DR in Saudi Arabia at the time.

Another study based on the same definitions of blindness and visual impairment was conducted in 1991 and reported a prevalence of blindness of 0.7% and visual impairment of 10.9%; among the visually impaired, only 1.3% had DR as an etiology [12]. At around the same time, a review article determined DR as a major cause of blindness in the Western world, and it was found responsible for 10-20% of cases in different studies and as the major cause of blindness among people of working age [13].

A more recent study has concluded that the prevalence of visual impairment among Saudis in 2005 was 13.9%, with DR causing 20.9% of cases. These figures, however, might be inflated because one of the limitations of this study was that 34.4% of participants were illiterate, 42.3% were unemployed, and 30.3% were above 50 years of age, all considered significant risk factors in the same study [14]. A retrospective study examined patients visiting a low-vision clinic in Riyadh during the period from February 2008 to June 2010, and set out to determine the causes of visual impairment in these patients. The study reported that 15.7% of patients had DR as the cause of their visual impairment [15].

These studies demonstrate the increasing prevalence of DR as a cause of visual impairment, and ultimately reflect the low prevalence of diabetes mellitus in Saudi Arabia in the past and its dramatic increase in recent decades.

A number of studies about the prevalence of diabetes mellitus in Saudi Arabia have been carried out since, with all of them showing the increasing rates of diabetes mellitus prevalence. The first study was published in 1982; it only included a small sample of rural Saudi men and determined the prevalence to be 2.5% among the population [16]. In another study in 1987, the prevalence of diabetes mellitus was found to be 4.3% [17]. This study too had the same flaws of including a small sample of rural subjects, although it studied both genders. Nonetheless, it clearly reflects the increasing number of Saudis affected by diabetes mellitus. Another study published in 1997 showed the continued increase in prevalence, with a higher prevalence in urban (12% in males and 14% in females) than rural populations (7% in males and 7.7% in females) [18].

In accordance with this trend, a more recent study on the prevalence of diabetes mellitus in Saudi Arabia that was conducted in 2009 reported the continuing increase of diabetes mellitus in Saudi Arabia, affecting 30% of the population [19]. This figure, however, could be slightly exaggerated since the study was not a population-based one and only included subjects attending primary healthcare centers, and these tend to be older patients with chronic diseases such as diabetes mellitus. Nevertheless, with the dramatically increasing prevalence of diabetes mellitus in Saudi Arabia, one should expect a similar increase in its complications, and DR is no exception to this.

In a study by Al-Rubeaan et al. analyzing the Saudi National diabetes registry [20], the results showed that 19.7% of the total 50,464 patients with diabetes enrolled in the study had DR, and a significantly higher proportion among males (61.2%) compared to females (38.8%); patients who had nonproliferative diabetic retinopathy (NPDR) comprised 9.1% of the total study population, while 10.6% of the sample had proliferative diabetic retinopathy (PDR) [20]. In another study conducted in the Al Hasa region of Saudi Arabia during the period from 2007 to 2009, DR prevalence was determined to be 29.4% among diabetic patients; 64.8% of these patients had mild NPDR, 23.9% had moderate NPDR, and only 2.8% had severe NPDR, while 7.8% had PDR [21].

Another study was conducted in Taif, Saudi Arabia, the goal of which was to find out the prevalence of blindness and visual impairment in 3,052 Saudi subjects. The reported prevalence of blindness (visual acuity of <3/60) was 2.6%, and that of bilateral severe visual impairment (visual acuity of <6/60 and ≥3/60) was 1.7%, while visual impairment (visual acuity of <6/18 and ≥ 6/60) constituted 7.5%. The prevalence of diabetes mellitus reported in this study was 29.7%; of those, 36.8% had DR, which was the etiology of 29% of blindness cases among diabetics, which translates into 10% of all blindness cases among the general population [22]. The age of participants (≥50 years) and the definition of diabetes mellitus (self-report of previous diagnosis, self-report of taking treatment to control blood glucose, or a random blood glucose level of >200 mg/dL) in this study might have led to slightly inflated results. This study used a different grading system (the Scottish Diabetic Retinopathy Grading Scheme); 22% of diabetic patients had mild DR, while 5% had observable DR; referable DR was seen in 3.9% of diabetics, and only 3.5% had PDR [22].

In another study that was carried out in Medina, Saudi Arabia, the results showed that 36.1% of diabetic patients had DR; of those, 37.7% had mild NPDR, 22% had moderate NPDR, and 22.4% had severe NPDR, with only 17.6% having PDR [23]. These results are very similar to the global prevalence of DR among diabetic patients as determined by Yau et al. in a pooled analysis of 35 different studies from around the globe (34.6%) [6]. In another study conducted in Jazan, Saudi Arabia [24], about 27.8% of diabetic participants had DR. This study, similar to the one in Taif [22], used the Scottish Diabetic Retinopathy Grading Scheme; 18% of all diabetic patients were found to have mild DR, 5.3% had observable DR, 3.5% had referable DR, and 1.1% had PDR [24]. These figures are lower than the reported figures in other studies from other regions of Saudi Arabia, which is odd considering the poor control of diabetes in those patients, as 60.3% of participants in this study had a blood glucose of >200 mg/dL (>11.1 mmol/L), reflecting a poor control of diabetes. However, it should be noted that Jazan is a region with a relatively healthy agrarian community [24], which may contribute to the lower complications of diabetes mellitus among its population. This issue, however, needs more research.

A study conducted in Abha [25], Saudi Arabia, reported a DR prevalence of 36.4% among 401 patients with type 2 diabetes mellitus. Of note, 57.5% of those patients had mild NPDR, 19.9% had moderate NPDR, and severe NPDR was noted among 11% of the patients, whereas 11.6% had PDR. Exudative and focal maculopathy were evident in 7.2% of patients [25]. A recent study conducted by Magliah et al. in Jeddah, Saudi Arabia [26], reported a three-year screening prevalence in the primary care setting among 250 patients, The results showed a prevalence of 15.2% initially, 19.6% at one year, 22.4% at two years, and 25.6% at three years. The prevalence of mild NPDR initially, at one year, at two years, and at three years was 9.6%, 14%, 16.0%, and 16.8%, respectively. The prevalence of moderate NPDR was 4% initially and at one year, 4.8% at two years, and 5.6% at three years. At the third year, only 0.8% of patients had severe NPDR. As for maculopathy, the results were the same initially, at one year, and at two years with 1.6%, which increased to 2.4% at three years. The annual increase in mild, moderate, and severe NPDR and maculopathy denotes the importance of regular screening as the severity progresses annually [26].

In a cross-sectional study conducted by Parrey et al. in Arar, Saudi Arabia [27], DR was identified as the third leading cause of visual impairment after cataract and refractive errors. The authors followed the WHO classification of visual impairment. Out of a total of 166 cases, 22 were found to have DR (13.2%) [27]. Similar results were reported from a study done by Al-Shaaln et al. in Aljouf, Saudi Arabia [14]. In this study, DR was the third most common cause of visual impairment following refractive errors and cataracts, with a prevalence of 20.9%. However, it was the main cause of visual impairment among patients aged 50-59 years and among those who had chronic diseases, with a prevalence of 48.1% and 39.1% respectively [14].

In another cross-sectional study done by Yasir et al. in Riyadh Governorate (excluding the capital city), Saudi Arabia [28], the authors reported a DR prevalence of 44.7%. The severity of DR was graded according to the Early Treatment of Diabetic Retinopathy Study (ETDRS). Diabetic macular edema (DME) was not incorporated with the DR classification. Sight-threatening diabetic retinopathy (STDR) includes PDR and/or DME only. Out of the examined 395 cases, 49 (12.4%) had STDR. The details of the prevalence according to the mentioned classification are as follows: no DR (66%), NPDR (21%), NPDR and DME (8%), PDR (2%), PDR and DME (2%), and DME (1%) [28].

In another more recent study carried out by Alramadan et al. in three major cities (Riyadh, Jeddah, and Hafouf) in Saudi Arabia in 2019 [29], the risk factors for macro- and micro-vascular complications of diabetes mellitus were addressed. The results showed a DR prevalence of 42.8% [29]. In a study conducted by Huda Alghamdi in Al Baha, Saudi Arabia [30], the causes of irreversible unilateral or bilateral blindness were addressed. DR was identified as the most common cause of blindness. Out of a total of 100 patients, 30 had DR (30%) [30]. This study, however, had a relatively small sample size; it also excluded patients with treatable conditions such as cataract or refractive errors. Table 1 summarizes the studies about the prevalence of DR conducted in Saudi Arabia from 2010 to 2019.

**Table 1:** Studies about the prevalence of DR in Saudi Arabia, 2010-2019 DR: diabetic retinopathy; NPDR: nonproliferative diabetic retinopathy; PDR: proliferative diabetic retinopathy; DME: diabetic macular edema

Study	Year	Sample population	Prevalence of DR among diabetics	Prevalence of mild NPDR	Prevalence of moderate NPDR	Prevalence of severe NPDR	Prevalence of PDR	Comments
Khan et al. (Al Hasa) [21]	2010	527	29.4%	64.8% of total DR	23.9% of total DR	2.8% of total DR	7.8% of total DR	N/A
Al Ghamdi et al. (Taif) [22]	2012	3,052	36.8%	22% among diabetics	Observable DR 5% among diabetics	Referable DR 3.9% among diabetics	3.5% among diabetics	The study used the Scottish DR grading scheme
El-Bab et al. (Medina) [23]	2012	690	36.1%	37.7% of total DR	22% of total DR	22.4% of total DR	17.6% of total DR	N/A
Al-Rubeaan et al. [20]	2014	50,464	19.7%	Overall prevalence of NPDR: 9.1%			10.6%	Registry-based study
Hajar et al. (Jazan) [24]	2015	3,659	27.8%	18% among diabetics	Observable DR 5.3% among diabetics	Referable DR 3.5% among diabetics	1.1% among diabetics	The study used the Scottish DR grading scheme
Ahmed et al. (Abha) [25]	2016	401	36.4%	57.5% of total DR	19.9% of total DR	11% of total DR	11.6% of total DR	N/A
Magliah et al. (Jeddah) [26]	2018	250	25.6%	16.8% of total DR	5.6% of total DR	0.8% of total DR	2.4% of total DR	3-year screening interval study
Yasir et al. [Riyadh Governorate (except capital)] [28]	2019	395	44.7%	Overall NPDR prevalence is 29% of total DR			4% of total DR	DME is mentioned separately (11%)

Risk factors for diabetic retinopathy

In the study by Al-Rubeaan et al. [20], the duration of diabetes mellitus for ≥10 years and older age were the most significant risk factors for DR. The prevalence of DR had a 50-fold increase between the youngest age group with shortest diabetes duration (25-44 years, <5 years) and the oldest group with the longest duration (≥65 years, ≥15 years) [20]. This is consistent with other studies that have reported the prevalence of DR along with its risk factors [21,23,25,26,28,29].

In the study done by Alramadan et al., advancing age was found to be significantly associated with DR. The prevalence in patients aged ≤60 years, 61-70 years, and ≥71 years was 38.5%, 46.8%, and 55.4% respectively [29]. A significant association with physical inactivity was also observed [29]. In the study by Alramadan, the reference for physical inactivity was <150 min/week. The prevalence of DR in patients who were physically active compared to inactive patients was 31.4% and 47.6% respectively (p: <0.001). The odds of having DR was increased by 60% (p=0.002) [29].

Al-Rubeaan et al. have reported that the male gender was significantly associated with DR, with males having a prevalence of 61.2% compared to 38.8% for females [20]. This is in agreement with the Medina and Riyadh Governorate studies [23,28], but in contrast with the studies done in Al Hasa [21], Abha [25], and Jeddah [26] regions, and with the study by Alramadan et al. [29] who reported no significant correlation between gender and DR.

The presence of other diabetic complications, especially nephropathy, was significantly associated with developing DR as reported by Al-Rubeaan et al. [20]. In the study done by Magliah et al., 44.7% of DR patients had nephropathy (p=0.0005) [26], however, this was not deemed a significant risk factor in the Abha study [25]. Other complications of DM were significantly associated with DR in the study that was done in Medina [23].

The use of insulin and poor glycemic control were also found to be significant risk factors for DR [20,21,26]. Alramadan et al. have reported that patients on insulin therapy were 90% more likely to develop DR (OR=1.9, p: <0.001) [29]. The study done in Abha showed a significant correlation between insulin use and DR, but there was no significant association with poor glycemic control [25]. The other studies did not report any correlation, but since insulin use is generally associated with advanced diabetes, one would presume a positive correlation anyway. The Medina study showed conflicting reports of not considering poor glycemic control as a risk factor but stating at the same time that high HbA1C was associated with DR [23].

Hypertension is also a significant risk factor for DR, with more than 50% of DR cases having hypertension as reported by Al-Rubeaan et al. [20], which is in contrast with the studies done in Abha [25] and Al Hasa [21], but in agreement with the study done in Medina [23] and the study conducted by Alramadan et al. [29]. Moreover, hypertension was reported in 78.9% of DR cases in a recent study by Magliah et al. [26].

Al-Rubeaan et al. have reported that smoking and hyperlipidemia did not appear as significant risk factors for DR [20]; the study in Abha supports these reports but found hyperlipidemia to be significant in severe cases of DR [25]. The study done by Alramadan et al. found no significant association between smoking or hyperlipidemia and DR [29]. On the other hand, The study done in Medina showed that both smoking and hyperlipidemia have a positive association with DR [23], while the Al Hasa study reported hypercholesterolemia as a significant risk factor, with no record related to smoking status available in the study [21]. In the recent study of a three-year screening interval that took place in Jeddah, smoking was not associated with DR (p=0.992), while hyperlipidemia was found to have a significant association (p=0.002) [26].

A family history of diabetes mellitus has also been addressed as a risk factor. Patients who have a family history of diabetes mellitus are 50% more likely to develop DR (OR=1.5, p=0.01). Also, a lower level of education seems to be significantly associated with DR (p: <0.001). However, these associations were only reported in the Alramadan study [29].

Body mass index (BMI) was also found to be significantly lower in patients with DR compared to diabetics without DR [22,23,27]; however, the Medina [23], Jeddah [26], and the Alramadan study [29] did not show any significant association between BMI and DR.

Awareness of diabetic retinopathy among diabetic patients

Studies have shown that patients’ knowledge and awareness of their diabetes is crucial for good glycemic control and lowering the risk of complications [31], including DR. A lack of awareness also contributes to poor compliance by patients [32].

A study was conducted at King Abdulaziz University Hospital to assess patients’ awareness of DR. Of the 357 diabetic patients involved in the study, 61% were aware of DR. There was a significant difference between patients with type 1 and type 2 diabetes regarding the level of awareness (63.8% vs. 36.2% respectively). Even though almost two-thirds of patients were aware of DR, only 50% had ever had an eye checkup, and only 19.8% were undergoing regular annual checkups [33].

An assessment of the awareness of preventive measures has also been carried out; 61.9% of diabetics considered the use of diabetic medications as a preventive measure against developing DR, 54% mentioned following a diabetic diet, 32.2% stated maintaining good glycemic control, and 22.4% mentioned maintaining good blood pressure [33].

In one study done by Mashaer Fallatah in Jeddah, Saudi Arabia [34], 92.4% of the participants had a satisfactory level of awareness, but only 10.5% knew about the frequency of regular eye screening. Moreover, the author assessed participants’ awareness about the complications of diabetes including DR, and a significant association was found with the level of education, sources of information, and the area of residency. Patients with a higher level of education had better knowledge about complications and vice versa (p: <0.05). Also, participants who received their information from mass media had better awareness compared to those who got their information from other sources (healthcare workers, friends, and relatives) (p: <0.05). Participants who lived in Jeddah exhibited a greater level of awareness than those from outside the city. This study, however, was conducted in a specialized eye hospital, and its findings may not be representative of the general public [34].

Another important aspect in the diagnosis and prevention of DR is patients’ adherence to recommended screening. In the study conducted by Alwazae et al. in Riyadh, Saudi Arabia [35], barriers to screening were the focus of the study. The study included 404 participants, and 20% of them had DR. Fear of the results, cost of screening, and lack of family support were reported among the participants. However, these factors were not significantly different between those who attended the screening and those who did not. Two factors were significantly associated with non-attendance: a lack of information about the screening process and participants’ belief that screening is not effective (p: <0.001 and p=0.002 respectively). On the other hand, adequate knowledge, longer duration of DM, and the presence of neurological complications increased adherence by 5%, 179%, and 114% respectively [35].

These results indicate the importance of implementing educational programs for diabetic patients to increase their awareness and level of self-efficacy. In a meta-analysis of 31 randomized controlled trials (RCT) involving a total of 3,731 patients and providing evidence for the effectiveness of diabetic self-management education (DSME) for patients with type 2 diabetes mellitus on their glycemic control, it was concluded that DSME decreases HbA1c by an average of 0.76% at immediate follow-ups; it was also found that there was a 1% decrease in HbA1c with every 23.6 hours of education [36]. These improvements are clinically significant since every 1% reduction in HbA1c over 10 years accounts for 21% reduced risk for any endpoints related to diabetes, 21% for deaths attributed to diabetes, 14% lower risk for myocardial infarctions, and 37% for microvascular complications [37].

## Conclusions

DR is a serious complication of diabetes mellitus. It is also a leading cause of blindness worldwide. The prevalence of DR in Saudi Arabia has risen substantially in recent decades, as a direct consequence of the dramatic increase in the prevalence of diabetes mellitus. Significant risk factors for DR include older age, longer duration of diabetes, poor glycemic control, and hypertension. Awareness and education about diabetes mellitus are associated with better outcomes and fewer complications.
